# Characterization of genomic alterations in Chinese colorectal cancer patients with liver metastases

**DOI:** 10.1186/s12967-021-02986-0

**Published:** 2021-07-19

**Authors:** Hong-Wei Wang, Xiao-Luan Yan, Li-Jun Wang, Meng-Huan Zhang, Chun-He Yang, Wei-Liu, Ke-Min Jin, Quan Bao, Juan Li, Kun Wang, Bao-Cai Xing

**Affiliations:** 1 Hepatopancreatobiliary Surgery Department I, Key Laboratory of Carcinogenesis and Translational Research, Ministry of Education, Peking University School of Oncology, Beijing Cancer Hospital and Institute, Haidian District, Beijing, China; 2 GloriousMed Clinical Laboratory (Shanghai) Co., Ltd, Shanghai, China

**Keywords:** Colorectal cancer, Liver metastases, Genomic alterations, Heterogeneity, Targeted sequencing

## Abstract

**Background:**

The exploration of genomic alterations in Chinese colorectal liver metastasis (CRLM) is limited, and corresponding genetic biomarkers for patient’s perioperative management are still lacking. This study aims to understand genome diversification and complexity that developed in CRLM.

**Methods:**

A custom-designed IDT capture panel including 620 genes was performed in the Chinese CRLM cohort, which included 396 tumor samples from metastatic liver lesions together with 133 available paired primary tumors.

**Results:**

In this Chinese CRLM cohort, the top-ranked recurrent mutated genes were *TP53* (324/396, 82%), *APC* (302/396, 76%), *KRAS* (166/396, 42%), *SMAD4* (54/396, 14%), *FLG* (52/396, 13%) and *FBXW7* (43/396, 11%). A comparison of CRLM samples derived from left- and right-sided primary lesions confirmed that the difference in survival for patients with different primary tumor sites could be driven by variations in the transforming growth factor β (TGF-β), phosphatidylinositol 3-kinase (PI3K) and RAS signaling pathways. Certain genes had a higher variant rate in samples with metachronous CRLM than in samples with simultaneous metastasis. Overall, the metastasis and primary tumor samples displayed highly consistent genomic alterations, but there were some differences between individually paired metastases and primary tumors, which were mainly caused by copy number variations.

**Conclusion:**

We provide a comprehensive depiction of the genomic alterations in Chinese patients with CRLM, providing a fundamental basis for further personalized therapy applications.

**Supplementary Information:**

The online version contains supplementary material available at 10.1186/s12967-021-02986-0.

## Background

Colorectal cancer (CRC) is one of the most common causes of cancer death worldwide [1], and distant metastases are often observed at diagnosis (synchronous metastases), with the liver being the most frequently affected organ [2]. Even if liver metastasis is not detected initially, there is a considerable probability that it will develop later (metachronous metastases). Perioperative chemotherapy combined with hepatectomy is expected to be the best treatment to cure colorectal liver metastasis (CRLM), but prognosis following resection of CRLM is still poor [3, 4]. There is an urgent need for prognostic and predictive biomarkers that can aid the selection of optimal neoadjuvant regimens and postoperative management strategies.

Many studies regarding the characterization of genomic alterations in metastatic colorectal cancer (mCRC) have been conducted in past years to discover therapeutic biomarkers. Targeted sequencing of mCRC samples at Memorial Sloan Kettering (MSK) Cancer Center revealed that varied survival by tumor laterality can be predicted by certain genes [5]. A large-scale integrated omics study in a Chinese CRC cohort demonstrated the ability of the phosphoproteome to distinguish metastasis and to predict drug response [6]. In research specifically focusing on CRLM, high similarities were observed between primary tumors and liver metastases (LMs), whereas ubiquitous private mutations in LMs suggested individual tumor heterogeneity and specific genetic biomarkers that were capable of predicting potential therapeutics for treating LMs [7–9]. However, these studies either handled primary tumors, LMs and other metastases identically or were restricted to quite small numbers of samples. To comprehensively characterize the genomic landscape of Chinese CRLM patients and explore potential prognostic biomarkers, we performed targeted sequencing of LM lesions and available paired primary tumors in a large Chinese CRLM cohort.

## Materials and methods

### Patients and samples

A total of 529 specimens from 396 patients with CRLM were collected from the Hepato-Pancreato-Biliary Surgery Department I, Peking University Cancer Hospital between January 2015 and October 2020, including 396 tissue samples from metastatic liver lesions and 133 paired primary tumor samples. Acquired samples were subjected to next-generation sequencing using a targeted panel assay as were blood samples obtained from all patients. Microsatellite instability was assessed for each patient. Information about the patient’s age, sex, primary tumor location and times to metastasis was collected by reviewing the medical records, and the data are summarized in Table 1. Detailed clinical data is in Additional file 1: Table S1. The study was reviewed and approved by the Ethics Committee of Peking University Cancer Hospital.

**Table 1:** Patient characteristics

	Primary tumors (N = 133)	Liver metastases (N = 396)
Age, median (range)	57 (32–78)	58 (13–80)
Sex		
Male	65.4% (87/133)	67.7% (268/396)
Female	34.6% (46/133)	32.3% (128/396)
Primary site		
Right	21.8% (29/133)	17.7% (70/396)
Left	78.2% (104/227)	82.3% (326/396)
Timing of metastasis		
Synchronous	82.0% (109/133)	72.2% (286/396)
Metachronous	18.0% (24/133)	27.8% (110/396)
MSI status		
MSI-H	1.5% (2/133)	1.3% (5/396)
MSS	98.5% (131/133)	98.7% (391/396)

### Targeted sequencing

DNA was extracted from formalin-fixed paraffin-embedded (FFPE) tissues and white blood cells using a QIAamp DNA FFPE Tissue Kit (Qiagen, Hilden, Germany) and a Blood Genomic DNA Mini Kit (Cwbiotech, Beijing, China). A custom-designed IDT capture panel (Integrated DNA Technologies, Coralville, IA) was employed to capture the coding regions of 620 genes (Additional file 2: Table S2). Captured DNA fragments were then used for library preparation and quantification guided by KAPA Hyper Prep protocols (Kapa Biosystems, Wilmington, MA), followed by purification with AMPure XP (Beckman Coulter, Brea, CA) and quantification using a Qubit™ dsDNA HS Assay Kit (Thermo Fisher, Waltham, MA). The final library was sequenced with the a NovoSeq 6000 platform (Illumina, San Diego, CA) with a minimum depth of  ×  500.

### Variant identification

The sequencing reads were aligned to a human reference genome (hg19) using Burrows-Wheeler Aligner (BWA) after trimming the adapters with Trimmomatic. Duplicated reads were flagged with Picard, and then reads were realigned using the Genome Analysis Tool Kit (GATK). Mutect2 and HaplotypeCaller were used to identify somatic mutations and germline mutations. FACTERA was used to identify genomic fusions and breakpoints. The filter condition is break support  ≥  5. Mutations were annotated with ANNOVAR. Based on the mutation filtering scheme of MSK-IMPACT (https://www.accessdata.fda.gov/cdrh_docs/reviews/DEN170058.pdf), hotspot mutations with allele depths above 8 and variant frequencies above 2% and non-hotspot mutations with allele depths above 10 and variant frequencies above 5% were retained as somatic mutations. Annotation of pathogenic mutations in ClinVar or truncating mutations of genes related to hereditary colorectal cancer was employed to detect germline mutations. Copy number variations (CNVs) were identified using GATK, and genes with copy number ratios below 0.6 or above 2 were subjected to CNV filtering. Tumor mutational burden (TMB) was defined as the number of nonsynonymous somatic mutations per million bases.

### Statistical analysis

Statistical analyses were performed with R v3.6.0. The prevalence of mutants was compared between different groups using the chi-square test. Student’s t test was performed to compare TMB and the percentage of shared mutations between paired primary tumors and LMs.

## Results

### Mutation landscape of CRLM

**Fig. 1:**
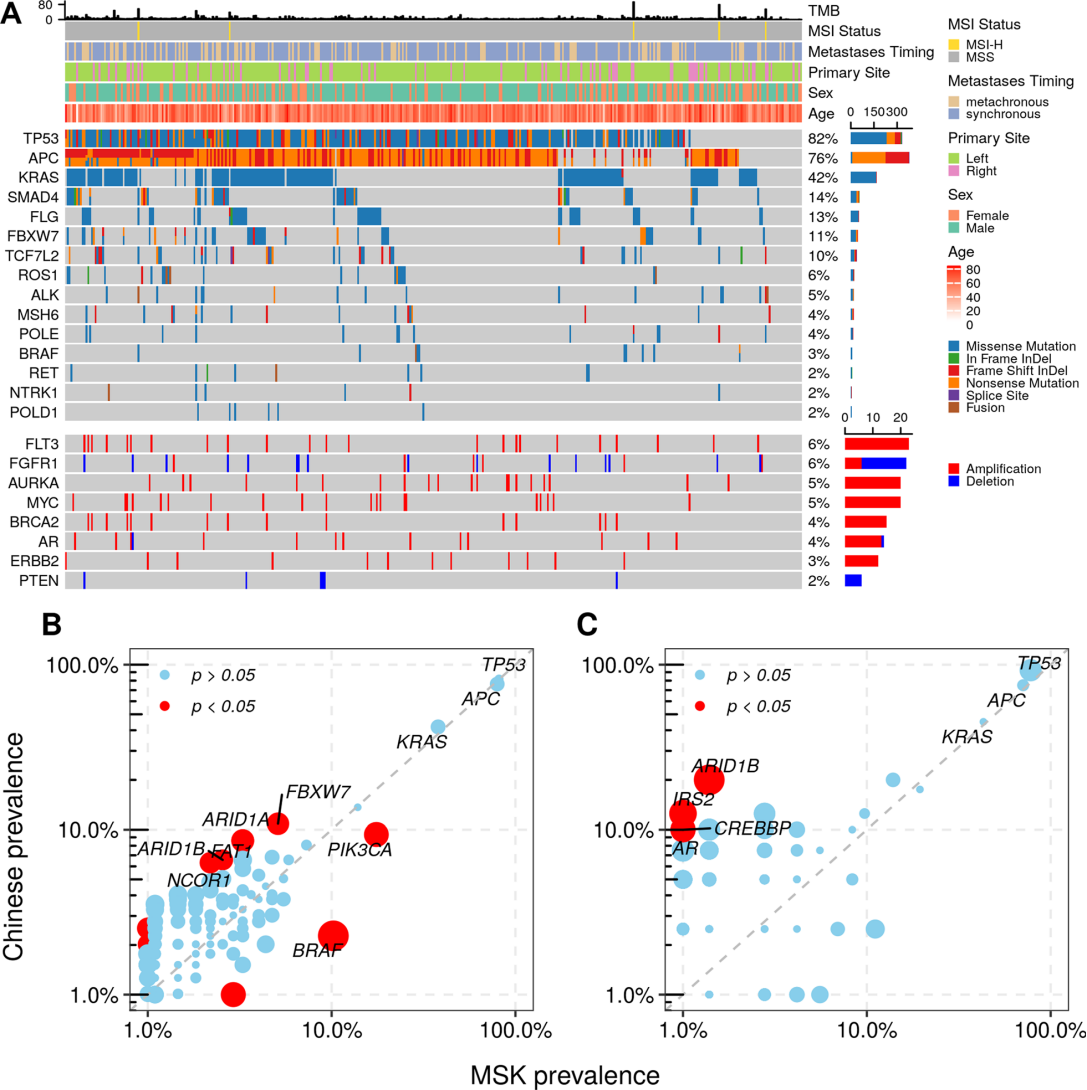
The genomic landscape of Chinese CRLM. **A** Recurrent mutations and CNVs in 396 Chinese patients with CRLM. The number and prevalence of altered genes are indicated on the right side of the heatmap. **B** Mutation prevalence of genes in Chinese and MSK CRLM cohorts. **C** Mutation prevalence of genes in treatment-naïve Chinese and MSK CRLM cohorts

In 396 metastatic liver samples, 7327 mutations in 620 genes were identified, including 6038 single nucleotide variants and 1289 small insertions/deletions, 20 germline mutations and 15 fusions (Fig. 1A). The top-ranked recurrently mutated genes observed in this cohort were *TP53* (324/396, 82%), *APC* (302/396, 76%), *KRAS* (166/396, 42%), *SMAD4* (54/396, 14%), *FLG* (52/396, 13%) and *FBXW7* (43/396, 11%). Germline mutations were detected in 20 samples (5.1%), and approximately one-third of the germline mutations (6/20) occurred in the *MSH6* gene. Druggable gene fusions were found in 2.8% of all samples (11/396), including 3 *ROS1*, 3 *RET*, 2 *ALK*, 2 *NTRK1* and 1 *BRAF* fusion. Somatic CNVs of 620 genes were also assessed, and fifty-two genes were determined to harbor such genomic events (Fig. 1A). The recurrent CNVs involved prominent amplification of the *FLT3*, *AURKA*, *MYC*, *BRCA2*, *AR* and *ERBB2* genes and dominant deletion of *FGFR1*, *FGFR3* and *PTEN*, each of which affected between 2 and 6% of all samples.

Mutation data of 274 LM samples were obtained by taking a subset of the mCRC cohort from MSK Cancer Center, and the data were downloaded from cBioPortal (https://www.cbioportal.org/study/summary?id=crc_msk_2017). To investigate the divergence of genomic alterations between different CRLM populations, the mutation rates were compared between the Chinese and MSK cohorts by assessing the somatic mutations of 337 (Additional file 2: Table S2) genes shared by our panel and MSK-IMPACT. Alterations in *PIK3CA* and *BRAF* were significantly enriched in the MSK cohort, while the Chinese cohort had a higher prevalence of mutations in genes like *FBXW7*, *FAT1*, *ARID1A*, *ARID1B* and *NCOR1* (Fig. 1B). Considering that the treatment conditions of the two cohorts were different, the frequencies of genic alterations were further compared considering only treatment-naïve patients (40 in the Chinese cohort and 60 in the MSK cohort). Higher mutation rates in the Chinese CRLM were found in four genes, *ARID1B*, *IRS2*, *AR* and *CREBBP* (Fig. 1C), and only *ARID1B* was in the overlap with differentially mutated genes of all samples.

### Genomic alterations by primary tumor site

**Fig. 2:**
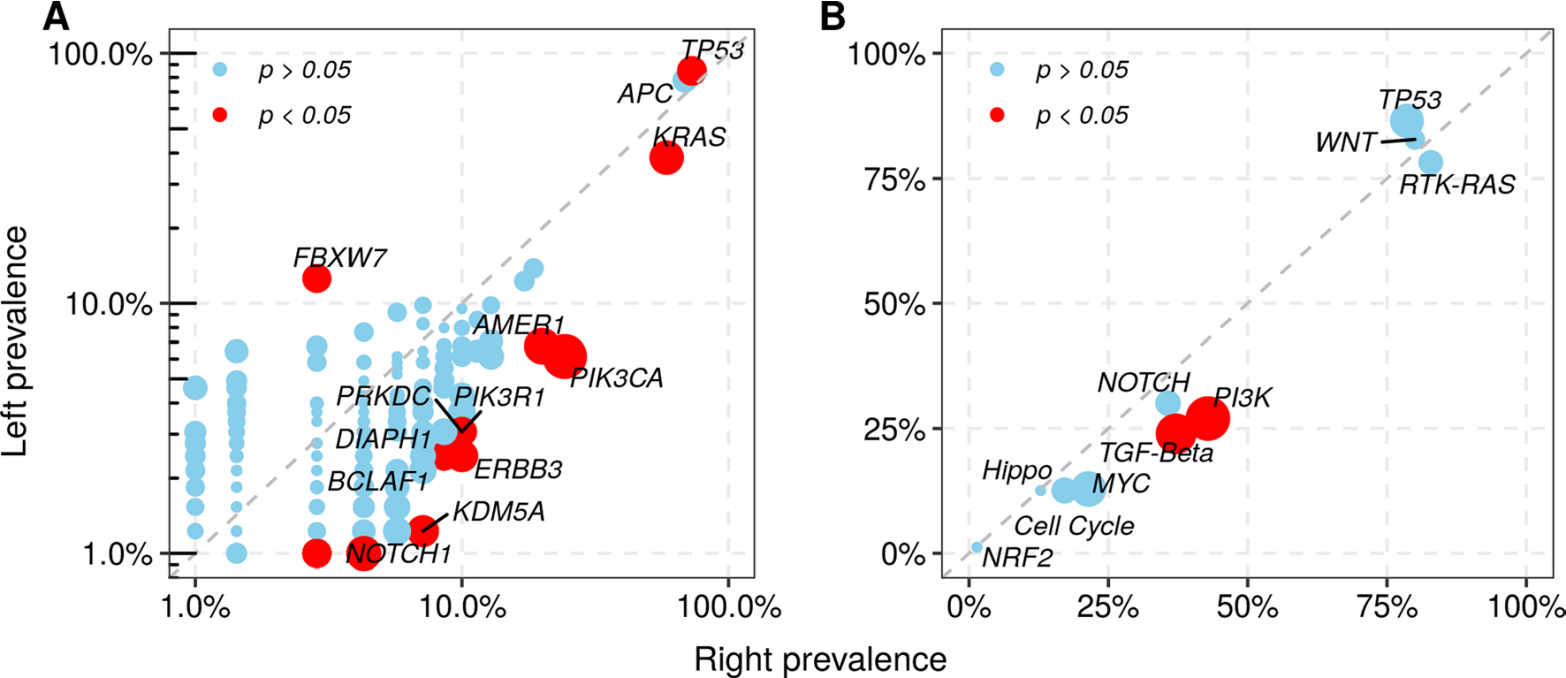
Genomic alterations by primary tumor site. **A** Gene-level mutation prevalence in liver metastases originating from left- and right-sided primary tumors. **B** Pathway-level mutation prevalence in liver metastases originating from left- and right-sided primary tumors. Significant elements are labeled as red circles

Recent studies suggest that shorter survival of mCRC patients with metastatic tumors originating from right-sided primary tumors is potentially caused by underlying features of somatic alterations [5]. We compared the frequencies of gene mutants in patients with different primary tumor sites. In addition to the previously reported genes *KRAS* and *PIK3CA*, other genes (such as *AMER1*, *PIK3R1*, *PRKDC*, *ERBB3*, *DIAPH1*, *BCLAF1*, *KDM5A* and *NOTCH1*) were also preferentially altered in LMs from right-sided primary tumors, while enrichment of alterations in *TP53* and *FBXW7* was observed in LMs originating from the left colon (Fig. 2A). Furthermore, comparative analysis of 10 oncogenic pathways derived from TCGA PanCancer Atlas between right- and left-sided primary sites was also conducted. Two pathways were significantly enriched in right-sided primary tumors, the transforming growth factor β (TGF-β) and phosphatidylinositol 3-kinase (PI3K) signaling pathways (Fig. 2B). Although the difference in mutation rates of RTK-RAS between the two primary site groups did not reach a significant level (82.9% vs. 78.2%, p  =  0.48), RAS showed more mutations in the right-sided primary tumors, while the RTK pathway did not (Additional file 4: Figure S1), and these results are consistent with a previous study [5].

### Genomic alterations according to time to metastasis

**Fig. 3:**
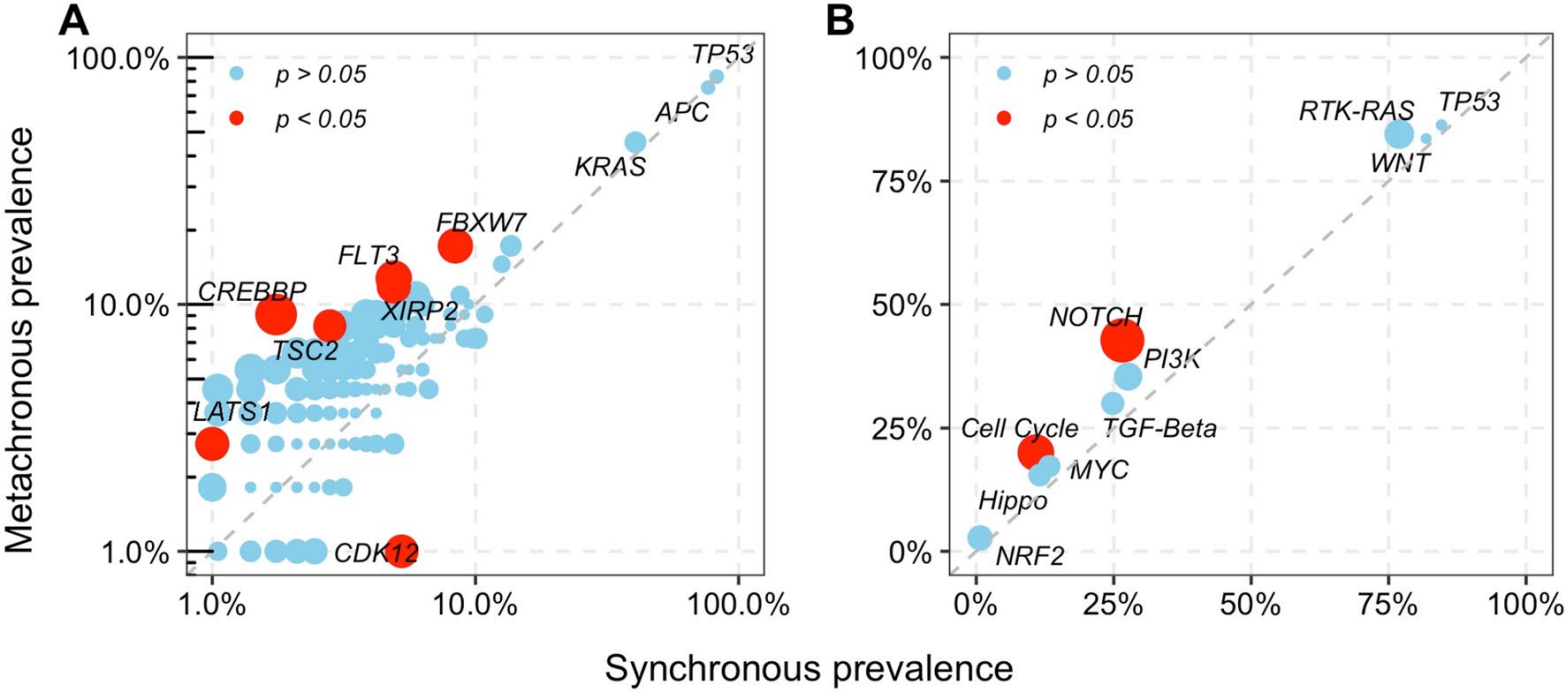
Genomic alterations by the timing of metastasis. **A** Gene-level mutation prevalence in synchronous and metachronous liver metastases. **B** Pathway-level mutation prevalence in synchronous and metachronous liver metastases. Significant elements are labeled as red circles

It has been reported that synchronous CRLM is correlated with poor survival, but the prognostic role of metastasis timing in CRLM remains controversial [10–12]. To clarify this contentious issue from the perspective of genomic alterations, we examined whether there is a differential distribution of genomic alterations between patients with different times to metastasis. Our results showed that mutations in *FBXW7*, *FLT3*, *XIRP2*, *TSC2*, *LATS1* and *CREBBP* were enriched in metachronous LMs, while only mutations in *CDK12* were enriched in synchronous LMs (Fig. 3A). Beyond the associations at the gene level, comparative analysis at the pathway level revealed that the Notch and cell cycle pathways were also selectively altered in metachronous CRLM (Fig. 3B). Additionally, a higher TMB in metachronous LMs than in synchronous LMs was also observed (Additional file 5: Figure S2).

### Mutations in primary tumors and liver metastases

**Fig. 4:**
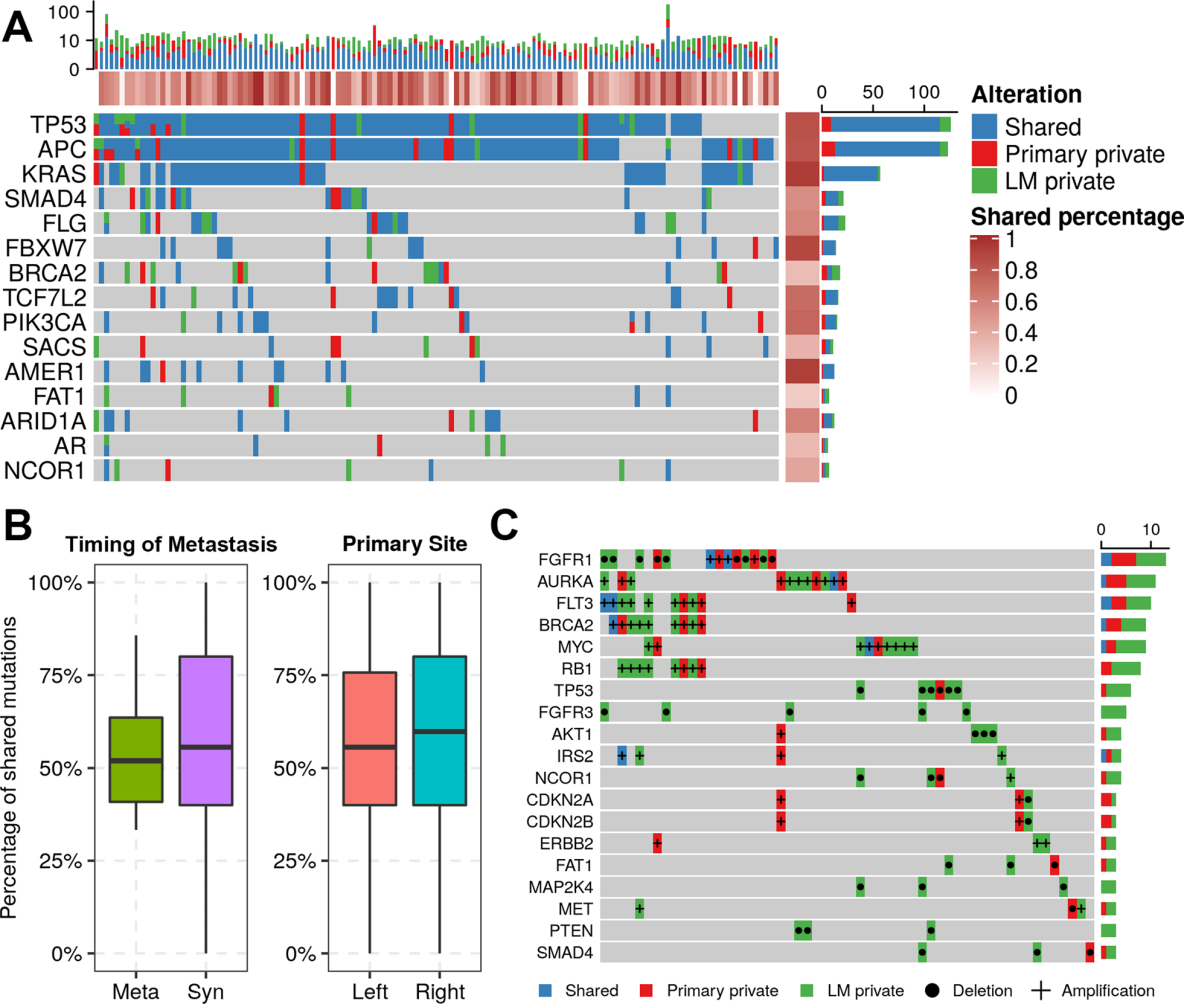
Concordances of genomic alterations between paired primary tumors and liver metastases (LMs). **A** Shared and private variants of recurrently mutated genes in 133 Chinese CRLM patients. The percentage of shared alterations for samples and genes is indicated on the top and right side of the heatmap. **B** Comparison of shared mutations between different primary tumor sites and the timing of metastasis. **C** Shared and private CNVs in Chinese CRLM patients

The concordance of mutations before and after metastasis was assessed in 133 patients with paired primary tumors and LMs (Additional file 3: Table S3). Shared mutations accounted for 56.7% of all mutations (951/1,685), while 19.7% (330/1,685) were private in primary tumors and 23.6% (399/1,685) were identified only in LMs. The median percentage of shared mutations among all detected genes was 50.0%. This number rose to 72.0% when focusing on the top 10 recurrently mutated genes (Fig. 4A). In particular, the proportions of shared mutations in three genes with the highest mutation rate all exceeded 80% (*TP53*: 83.5%, *APC*: 81.9%, and *KRAS*: 93.0%). No significant difference in shared mutations was observed between left- and right-sided primary tumors or samples from patients with different times to metastasis (Fig. 4B).

It is noteworthy that the occurrence of shared variants was much lower when only considering CNVs (Fig. 4C). The shared CNVs covered no more than 10% of all CNVs (13/148), and most of them were only in LMs (96/148, 64.9%). Specifically, several genes with only private deletions were observed in the metastases, including *FGFR3*, *MAP2K4* and *PTEN*.

## Discussion

With extensive research on the genomic characteristics of mCRC, the associations between mutations and the prognosis of patients have gradually been disclosed, and the correlations between primary tumors and LMs have been mined. However, in-depth investigation of genomic alterations of CRLM tumors from Chinese patients has not been performed. In this study, we collected a sizable number of eligible samples and identified comprehensive genomic alterations to explore the differences between patients with different characteristics.

The genomic landscape of Chinese patients with CRLM resembled that previously reported for mCRC [5, 6, 13], and *TP53*, *APC* and *KRAS* were the most frequently mutated genes [14, 15]. Alterations of another recurrently altered gene, *FLG,* were distributed throughout the entire gene, and most mutations were privately enriched in primary tumors or metastases, indicating that they were passenger mutations that emerged with tumor progression. It has also been confirmed that such variants fail to serve as prognostic indicators for CRC [16]. Published studies have shown that highly frequent mutations in members of the ARID gene family are associated with microsatellite instability [17]. Four out of five microsatellite instability-high patients had such mutations in our cohort. Compared to the MSK cohort, the Chinese cohort showed significantly more mutations of the ARID gene family, regardless of whether all samples or only treatment-naïve patient samples were analyzed. Both genetic and environmental factors can contribute to these disparities, and further validation may be warranted.

Recent studies have confirmed the prognostic role of primary tumor location in mCRC [18, 19] and its association with underlying genomic alterations [5]. In our cohort, the differential distribution of variants in the TGF-β, PI3K and RAS pathways based on primary tumor site was consistent with existing research. Apart from *KRAS* and *PIK3CA*, other genes preferentially altered in the right-sided primary tumor sites, such as *PRKDC*, *ERBB3*, *BCLAF1* and *NOTCH1*, have been reported to be correlated with poor prognosis or migration in CRC [20–26]. In our study, alterations in *FBXW7* were enriched in LMs derived from left-sided CRCs. Since a previous study found that patients with mutations in *FBXW7* may be less likely to respond to anti-EGFR therapy than those without mutations in *FBXW7* [12], *FBXW7* assessment may be useful to include in molecular testing of patients with left‐sided CRC who would receive targeted therapy.

Compared with left-sided primary tumors, right-sided primary tumors showed a higher proportion of synchronous LMs in our cohort (30.4% vs. 15.7%). Synchronous lesions were once expected to predict worse survival than metachronous lesions in hepatic metastases of CRC, but recent studies have expressed controversial opinions [27]. Interestingly, biomarkers that indicate a worse prognosis for mCRC, such as *FLT3* amplification, alterations of *FBXW7* and high TMB [28, 29], were found to be enriched in metachronous lesions in our cohort. Consequently, further confirmatory work is required to determine the prognostic value of the timing of metastasis.

Just over half of all genomic alterations were detected in both paired primary tumor and metastasis samples in our cohort. However, when only focusing on recurrent driver genes, this proportion increased to a level that was similar to that seen in several small CRLM cohorts (approximately 70%) [7, 8, 30, 31]. This change in proportion may be because systemic spread of colorectal cancer may occur while the tumor is clinically undetectable [31]. A high percentage of private CNVs in LM was observed in our cohort, which might have been caused by the intratumoral evolution of metastatic clones because metastases were more genetically similar in terms of copy number-based phylogenies [32]. A high CNV burden combined with *RAS*/*BRAF*^V600E^/*TP53* formed an efficient prognostic marker [33].

Although this work provides a comprehensive characterization of genomic alterations of liver metastatic tumors in a large Chinese CRLM cohort, there are limitations. This study was performed with samples from a single institution; therefore, the risk of selection bias cannot be completely ruled out. Without available clinical follow-up information, the prognostic effect of differential alterations in subgroups cannot be validated and was only supported by recent literature.

## Conclusions

In summary, we delineated a comprehensive genomic landscape of Chinese patients with colorectal liver metastases. Differences in alterations between patient subgroups were characterized, and heterogeneity of individual primary tumor and liver metastasis samples was unveiled. These findings could provide a fundamental basis for further personalized therapy applications.

## Supplementary Information

**Additional file 1: Table S1.** Detailed clinical data of the patients in the current study

**Additional file 2: Table S2.** The gene list of our targeted panel

**Additional file 3: Table S3.** The shared mutations between paired primary tumors and LMs

**Additional file 4: Figure S1.** Comparison of the mutation rates of RTK and RAS between primary tumor site

**Additional file 5: Figure S2.** The association between TMB values and the timing of CRLM diagnosis

## Data Availability

All data used during the study are available from the corresponding author by request.

## Abbreviations

CRC: Colorectal cancer
CRLM: Colorectal liver metastasis
CNVs: Copy number variations
LMs: Liver metastases
FFPE: Formalin-fixed paraffin-embedded
mCRC: Metastatic colorectal cancer
MSK: Memorial Sloan Kettering
PI3K: Phosphatidylinositol 3-kinase
TGF-β: Transforming growth factor β
TMB: Tumor mutational burden
