# Supplementary figures and images for: Characterization of genomic alterations in Chinese colorectal cancer patients with liver metastases
Source: J Transl Med. 2021 Jul 19;19:313. doi: 10.1186/s12967-021-02986-0 (PMC8287676; doi:10.1186/s12967-021-02986-0)

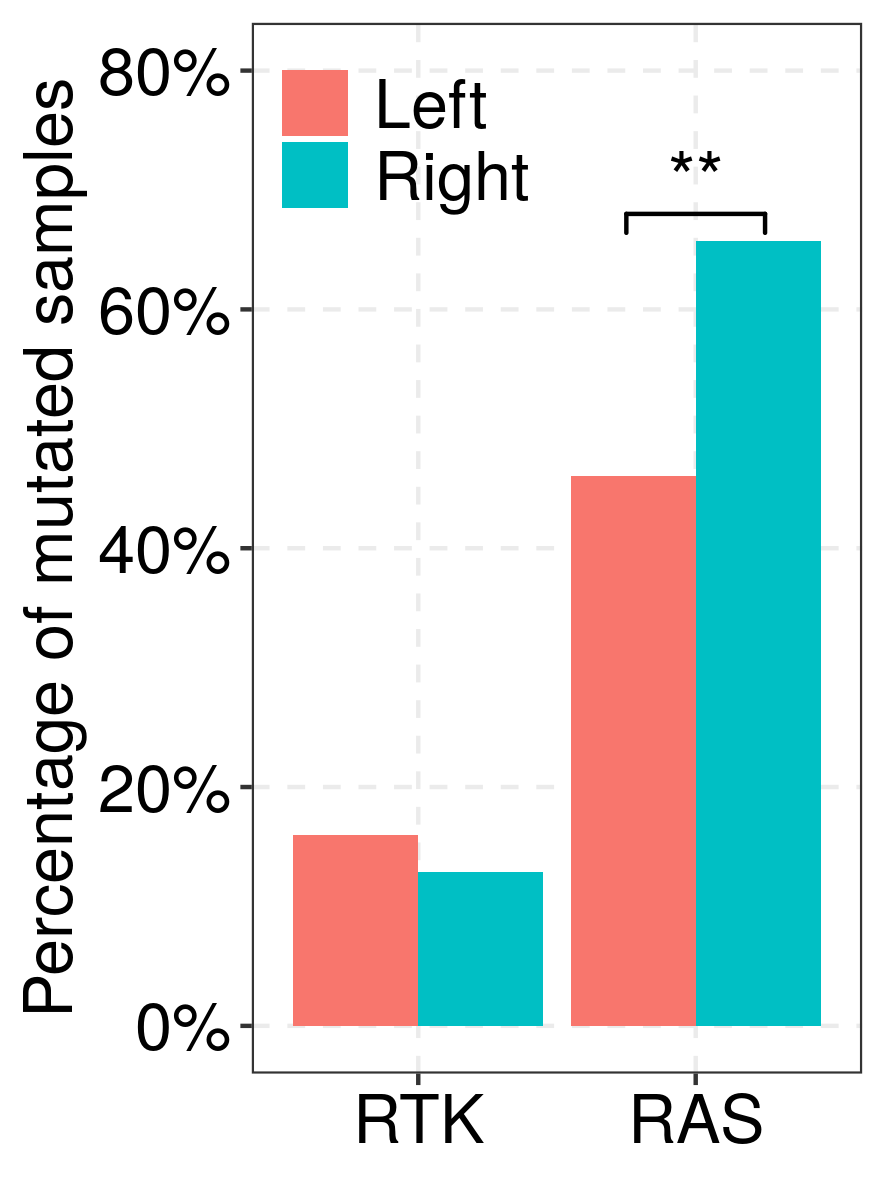

Supplement: Supplementary file 4 — Additional file 4: Figure S1. Comparison of the mutation rates of RTK and RAS between primary tumor site [file 12967_2021_2986_MOESM4_ESM.tif]

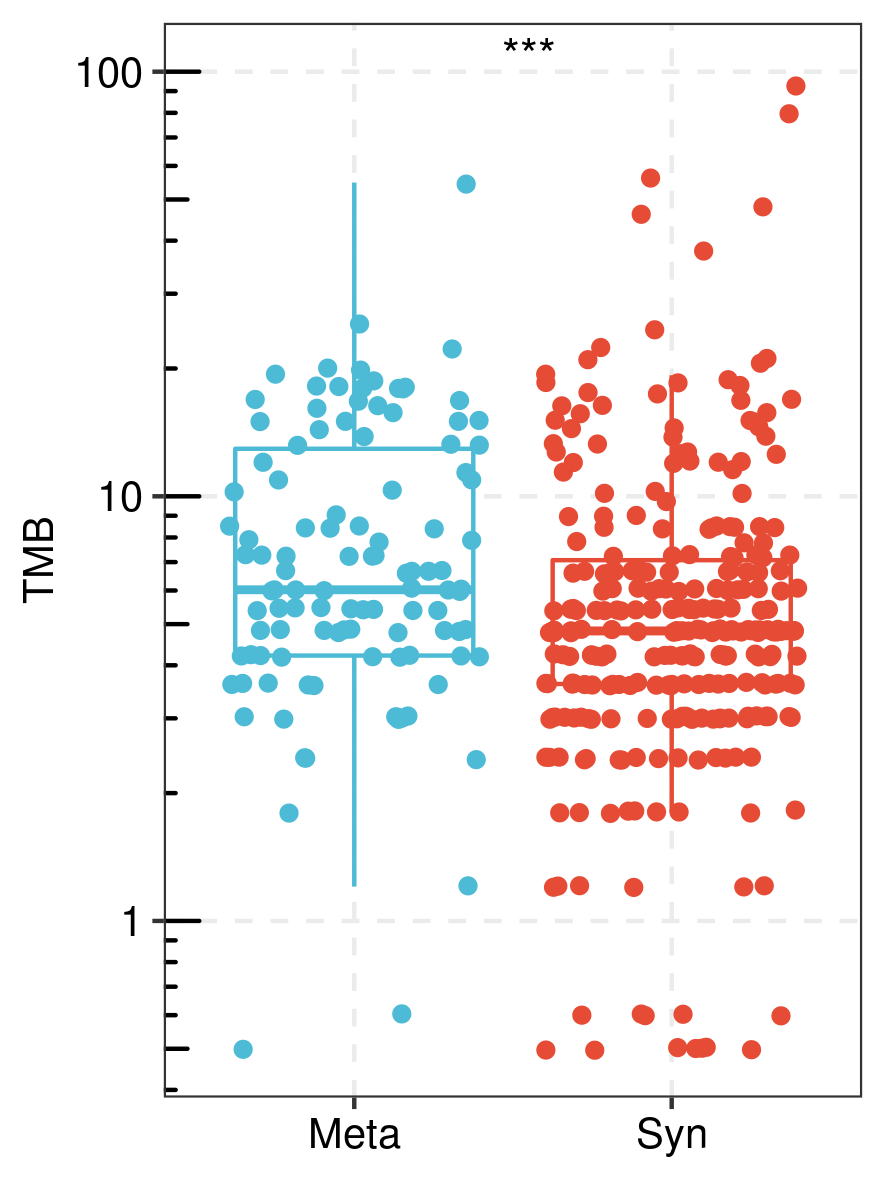

Supplement: Supplementary file 5 — Additional file 5: Figure S2. The association between TMB values and the timing of CRLM diagnosis [file 12967_2021_2986_MOESM5_ESM.tif]
